# Neonatal Screening for Sickle Cell Disease in Belgium for More than 20 Years: An Experience for Comprehensive Care Improvement

**DOI:** 10.3390/ijns4040037

**Published:** 2018-11-27

**Authors:** Béatrice Gulbis, Phu-Quoc Lê, Olivier Ketelslegers, Marie-Françoise Dresse, Anne-Sophie Adam, Frédéric Cotton, François Boemer, Vincent Bours, Jean-Marc Minon, Alina Ferster

**Affiliations:** 1 Department of Clinical Chemistry, LHUB-ULB, Université Libre de Bruxelles (ULB) 322, Rue Haute, 1000 Brussels, Belgium; 2 Department of Hemato-Oncology Hôpital Universitaire des Enfants Reine Fabiola, Université Libre de Bruxelles (ULB) 15, av. J.J. Crocq, 1020 Brussels, Belgium; 3 Department of Laboratory Medicine CHR de la Citadelle, 1, Boulevard de la 12^ème^ Ligne, 4000 Liège, Belgium; 4 Department of Pediatric, University Hospital Liège, CHR de la Citadelle, 1, Boulevard de la 12^ème^ Ligne, 4000 Liège, Belgium; 5 Department of Human Genetics CHU Sart Tilman, Université de Liège (ULg) Domaine Universitaire du Sart Tilmant Bâtiment 35-B, 4000 Liège, Belgium

**Keywords:** sickle cell disease, neonatal screening program, registry, birth prevalence

## Abstract

Our previous results reported that compared to sickle cell patients who were not screened at birth, those who benefited from it had a lower incidence of a first bacteremia and a reduced number and days of hospitalizations. In this context, this article reviews the Belgian experience on neonatal screening for sickle cell disease (SCD). It gives an update on the two regional neonatal screening programs for SCD in Belgium and their impact on initiatives to improve clinical care for sickle cell patients. Neonatal screening in Brussels and Liège Regions began in 1994 and 2002, respectively. Compiled results for the 2009 to 2017 period demonstrated a birth prevalence of sickle cell disorder above 1:2000. In parallel, to improve clinical care, (1) a committee of health care providers dedicated to non-malignant hematological diseases has been created within the Belgian Haematology Society; (2) a clinical registry was implemented in 2008 and has been updated in 2018; (3) a plan of action has been proposed to the Belgian national health authority. To date, neonatal screening is not integrated into the respective Belgian regional neonatal screening programs, the ongoing initiatives in Brussels and Liège Regions are not any further funded and better management of the disease through the implementation of specific actions is not yet perceived as a public health priority in Belgium.

## 1. Introduction

At the World Health Organization, sickle cell disease has been recognized as a global public health problem [1]. The different migratory flows of recent decades have influenced the disease in European countries and in particular in Belgium [2]. According to our survey in 2007, there were approximately 400 patients (0.0036% of the total population) with sickle cell disease (SCD) living in Belgium. The implementation of a Belgian registry for SCD in 2008 allowed us to demonstrate that at the end of 2012, 469 SCD patients were regularly followed and registered by eight Belgian hospitals [3].

The benefit of neonatal screening for SCD has been established for many years [4]. Birth prevalence of this disease is not the only criterion for choosing to include sickle cell disease in the neonatal screening program, but in countries where birth prevalence of the condition is greater than 1:6000, this has been shown to be cost-effective [5]. However, in several European countries such as Belgium, neonatal screening for SCD is not part of the national neonatal screening program. Indeed, in Belgium, there are no national recommendations for sickle cell disease screening at birth and only local initiatives offer the benefit of early diagnosis to a small number of families. Following a successful Belgian screening in 2013 for two-thirds of neonates performed as part of a pilot study, it has been shown that the birth prevalence of SCD was 1:2329 [6].

In this context, this paper gives an update on the neonatal screening results for SCD and overall hemoglobinopathies in two different Belgian regions. In parallel with the increase in the number of sickle cell neonates born in Belgium, it also highlights the initiatives that have been conducted to improve the clinical care program.

## 2. Material and Methods

### 2.1. Neonatal Screening for Sickle Cell Disease

Neonatal screening for SCD began in five Brussels maternity wards in December 1994, but has been offered to all neonates in all maternity wards of the Brussels Region since 2004 (2004–2017: 310,053 neonates screened); screening began in one maternity ward in Liège Region in 2002 and was extended to 15 maternity wards in Liège Region in 2009 (East of Belgium; 2008–2017: 186,829 neonates screened). It is realized in three screening centers i.e., Brussels University Laboratory (LHUB-ULB), Centre Hospitalier Régional (CHR) de la Citadelle and Centre Hospitalier Universitaire (CHU) du Sart Tilman. The neonatal screening process is described in Table 1 and has been detailed previously [7,8,9]. Briefly, in LHUB-ULB and CHR de la Citadelle, liquid umbilical cord blood samples in EDTA were screened initially using an isoelectric focusing technique (IEF) (Perkin Elmer Life Sciences, Zaventem, Belgium) and since 2008, using a capillary zone electrophoresis (CZE) technique (Sebia Benelux, Vilvoorde, Belgium) [6]. In CHU du Sart Tilman, heel prick samples on filter paper were screened by tandem mass spectrometry (TMS) [7,8]. If a hemoglobin variant is detected, further analysis is performed on the same sample using high performance liquid chromatography (BioRad, Hercules, CA, USA) or DNA analysis [7,8,9]. If confirmed, a new sample is requested to use as a control.

For the three screening centers, reports of results concern all SCD and all minor and major forms of hemoglobinopathy detected by the technique (Table 1). The screening of SCD in a neonate is reported immediately to the local coordinator and the medical staff of the maternity ward concerned. A new sample is requested for diagnosis.

For clarity, all results were also compiled in one period, i.e., 2009 to 2017, that allows us to report all results obtained from the three screening centers.

After confirmation of sickle cell disease on a new sample and, if feasible, before leaving the maternity ward, the families of an affected neonate received counselling and were referred to a specialized healthcare center where an initial visit was scheduled. The reference centers ensured the monitoring of affected children. The comprehensive care program includes the education of the patient and parents on subjects such as the prevention of complications, lifestyle, and management of fever and pain. The priority is to offer comprehensive and integrated care from neonatal screening throughout childhood and beyond to prevent acute complications, to delay the onset of chronic organ damage, and to treat acute complications for all neonates diagnosed with SCD. It is coordinated by a pediatrician who has acquired special skills in the care of patients with SCD.

### 2.2. Belgian Network of Health Care Providers and Registry for Sickle Cell Patients

In 2006, a red blood cell (RBC) disorders committee within a scientific society (the Belgian Haematology Society (BHS)) called the BHS RBC Committee was created. To date, it consists of 25 partners working in 14 different Belgian hospitals. The main objective of this network was to improve health care for patients affected by a non-malignant hematological disease, and in particular SCD. It does not currently benefit from any operating subsidy. It was supported on a temporary basis by grants. The main actors are pediatricians, adult hematologists, nurses and clinical biologists. To offer a unique tool to monitor the evolution of the population with SCD and to collect information on the main SCD complications (in particular for neonates screened at birth), a registry has been set up in 2008 by this committee. The objectives and implementation of the Belgian registry has been detailed previously [2]. Briefly, the BHS RBC Committee administers a centralized Belgian registry of patients with SCD. Eight centers participated with patient registration. Without national support, but thanks to a grant, in 2018, an updated registry has been launched and today, 12 centers participate. The items in the database were the subject of a consensus within the BHS RBC Committee. These data are updated annually. They make it possible to evaluate the benefit of neonatal screening and the follow-up of the patients screened at birth. For data privacy reasons, the information can only be accessed by healthcare professionals.

### 2.3. Comprehensive Care Improvement Plan

In 2014, to improve health care at a national level, the BHS RBC Committee proposed to define a plan of action. As part of the Belgian plan for rare diseases, this was submitted to the health authorities.

## 3. Results

### 3.1. Neonatal Screening for Sickle Cell Disease

During 2009 to 2017, 396,894 neonates were screened. This represents approximately 33% of births in Belgium. Screening coverage is almost 100%, except in two Brussels maternity wards where it is 85%. Birth prevalence of SCD and likely heterozygosity for hemoglobin (Hb) S are reported in Table 2. One sickle cell child born in one of the maternity wards where screening is performed was reported as not having been screened i.e., FSD^Punjab^. The maternity ward has a screening coverage of around 85%. Most of the patients are homozygous for Hb S or compound heterozygous for Hb S and beta-thalassemia (210/246); 27/246 patients had a Hb SC disease.

The highest birth prevalence of neonates likely heterozygous for Hb S were observed for the two most recent years in both Brussels and Liège regions (Table 2). It is the most frequent hemoglobin variant observed in both regions that offer screening for SCD, with the most prevalent sickle cell disease being homozygosity for Hb S; β-thalassemia was observed only for two and seven neonates in the Brussels and Liège regions, respectively (Table 3).

### 3.2. Belgian Network of Health Care Providers and Registry for Sickle Cell Patients

At the end of 2012, 469 patients were registered in the Belgian registry of patients with SCD. In September 2018, 538 patients had been registered in the updated database; 285 were born in Belgium, of which 64% (182/285) benefited from the neonatal screening program for SCD and 53 did not (i.e., born in other regions than those covered by neonatal screening and at least one born in the Brussels region). Compared to data in 2012, this means a longer follow-up and an increase in the number of affected patients screened at birth (or not).

### 3.3. Comprehensive Care Improvement Plan

**Figure 1:**
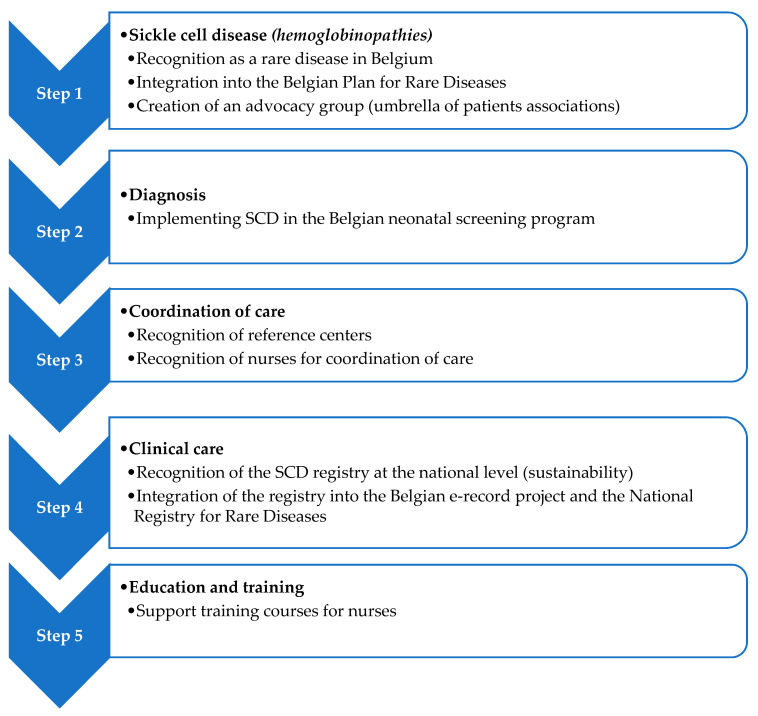
Plan of action to improve clinical care for SCD patients in Belgium.

A plan of action to improve health care for SCD patients has been proposed and submitted to the National Institute of Health in 2014 (Figure 1). To date, only the recognition of SCD as a rare disease in Belgium (Step 1) through the recognition of reference centers for hemoglobinopathies (Step 4) at the Belgian and European levels i.e., Cliniques Universitaires de Bruxelles Hôpital Erasme, and one related department i.e., Department of Hemato-Oncology, Immunology and Transplantation—Hôpital Universitaire des Enfants Reine Fabiola, has been effective.

The other steps of the plan (Figure 1) have not been debated or implemented. In particular, sickle cell disease has still not been included in the national neonatal screening program.

## 4. Discussion

Our study demonstrated that throughout the period of 2009 to 2017, the birth prevalence of sickle cell disease in two Belgian regions was higher than 1:2000 and heterozygosity for Hb S was the highest during the years 2016 and 2017 (>1:50 and >1:90 for Brussels and Liège Regions, respectively). To date, neonatal screening for SCD is not yet integrated into the national neonatal screening program. It also has no funding. Neonatal screening has been and is an important lever for providing health care improvements to sickle cell patients. In this respect, the creation of a Belgian network of health care professionals, particularly composed of many pediatricians, within a Belgian scientific society and the resulting initiatives (such as the implementation of a clinical registry of sickle cell patients) have been important steps. An action plan has also been proposed to the National Institute of Health in 2014.

In recent decades, as a result of migratory flows, the disease has spread throughout the world, particularly in Western Europe. It is therefore becoming a major concern of public health policies [10,11]. Belgium, with 20% of migrants including 12.5% of at-risk origin and its history of colonization in Africa and more particularly in the Democratic Republic of Congo, is also concerned [12]. During a pilot phase of neonatal screening extended over a period of six months and covering 2/3 of births in Belgium, the birth prevalence observed for SCD is 1/2329 [6]. This makes SCD one of the most common inherited diseases in Belgium, far more prevalent than other commonly screened disorders such as phenylketonuria. Birth prevalence of SCD remains at >1:2500 in regions were neonatal screening is performed. Despite the birth prevalence increases in 2016 and 2017 in the two regions where screening was performed, without national data, it is quite difficult to draw conclusions.

The advantage of neonatal screening in reducing early mortality is demonstrated in the US, various European countries and Jamaica. Vichinsky et al. showed that after a median follow-up of seven years, the overall mortality rate of patients diagnosed at birth was 1.8%, compared to 8% in children diagnosed after the age of three months [13]. Similarly, in the Jamaican cohort, less than 1% of children died in the first two years of life when preventive strategies were available, compared to 14% when early interventions could not be implemented [14]. In Brazil, however, the mortality rate of children with SCD remains high, at 7.4%, despite an effective, ongoing and comprehensive screening program. There are many reasons for this, including the low socio-economic and cultural status of affected families complicating regular clinical monitoring, long trips to health facilities, the short interval between onset of symptoms and death and inexperience of the health staff to recognize and manage SCD acute events [15]. Quinn et al. have demonstrated that neonatal screening minimizes morbidity and mortality through antibiotic prophylaxis and parental education [16,17]. Currently, with the addition of combined vaccination against pneumococcus and *Haemophilus influenzae* on the one hand, and integrated management that focuses on family education on the other hand, the mortality of newborns screened decreased to less than 1%. Screening is not widespread in Belgium, and in 2008, our BHS RBC Committee established a sickle cell registry. These two aspects allowed us to compare the future of SCD children that were screened (or not) at birth [18]. If we were unable to demonstrate a significant reduction in mortality among children screened in the neonatal period compared to those that were not, our previous results showed a benefit of neonatal screening in terms of decreasing the incidence of a first bacteremia and reducing the number and days of hospitalizations (expressed per 100 patient-years). Our only two deaths (in 1996 and 2000) occurred in very young patients, during a period marked by poor compliance and when integrated management was still immature [18]. Couque et al. also emphasized the importance of optimal adherence and integrated care on the prognosis of patients in 2016 [19]. The update of our cohort in the Belgian registry will allow us to monitor the effectiveness of the management of sickle cell patients and to provide any additional data regarding the benefit of screening.

The establishment of an integrated system for diagnosis and patient care is only beginning to emerge in Europe for several reasons. It concerns migrant minority communities of various origins, often isolated, disadvantaged and whose access to health care is not always easy; the perception of sickle cell disease in the communities concerned was not or is no longer adequate; and by lack of knowledge, the disease is often not recognized or trivialized by members of the medical community as well as by health managers [1]. In Belgium, a group of doctors involved in the management of sickle cell disease began in the early 2000s to reflect on the feasibility of an integrated care system initially in Brussels. This group wanted to focus on prevention while promoting health within a multidisciplinary group, as recommended by the World Health Organization [1]. This network aims to bring together all health professionals and patient associations concerned in a search for the best evidence of prevention, care and epidemiological data. It also aims to alert national health authorities to the real public health problems posed by SCD. This explains the approach that aims to propose an action plan for improving the management of SCD.

## 5. Conclusions

Neonatal screening for SCD performed in two Belgian regions demonstrated that its birth prevalence is higher than 1:2000. Those results pose the question of its integration into the regional neonatal screening program. In order to appreciate the benefit of neonatal screening in our care setting, a sickle cell registry has been set up and has been updated in 2018 with an increasing number of patients screened for ongoing evaluation. These initiatives have been one of the driving forces behind the creation of a Belgian network of healthcare professionals that aims to improve the management of all sickle cell patients.

## Figures and Tables

**Table 1:** Neonatal screening process (offered to all neonates). CHR = Centre Hospitalier Régional. IEF = isoelectric focusing. CZE = capillary zone electrophoresis. TMS = tandem mass spectrometry.

Screening Centre	Sample	First Screening Test	Confirmation Test (Same Sample)	Report of Results	Ref.
Brussels Region	Umbilical cord blood liquid	IEF (<2008) CZE (≥2008)	HPLC	Any hemoglobinopathy detected	[7]
CHR Citadelle	Umbilical cord blood liquid	CZE	HPLC	Any hemoglobinopathy detected	[8]
Liège Region	Heel prick/filter paper	TMS	DNA	Any hemoglobinopathy detected	[9]

**Table 2:** Distribution by year of neonates screened as having a sickle cell disease (SCD) i.e., homozygous for Hb S, compound heterozygous for Hb S and β-thalassemia, Hb C or another Hb variant; or likely heterozygous for hemoglobin S (FAS).

Year	Neonates Screened	SCD	SCD	FAS	FAS	FAS	FAS
	All Regions	Both Regions	Both Regions	Brussels Region	Brussels Region	Liège Region	Liège Region
	(*n*)	(*n*)	Birth Prevalence	(*n*)	Birth Prevalence	(*n*)	Birth Prevalence
**2009**	40,026	25	1:1601	421	1:54	159	1:109
**2010**	40,579	25	1:1561	449	1:52	150	1:115
**2011**	40,262	36	1:1088	458	1:50	112	1:154
**2012**	40,675	24	1:1768	460	1:51	175	1:98
**2013**	40,241	22	1:1829	520	1:45	161	1:104
**2014**	40,144	28	1:1487	447	1:52	192	1:87
**2015**	39,748	27	1:1529	449	1:52	174	1:94
**2016**	39,292	25	1:1572	504	1:46	200	1:82
**2017**	37,364	35	1:1068	503	1:42	193	1:83
**Total**	358,331	251	1:1427	4211	1:49	1516	1:100

**Table 3:** Neonatal screening for SCD by region: 2009–2017.

Type	Birth Prevalence	Birth Prevalence
	Brussels Region	Liège Region
	*n* = 206,984	*n* = 151,347
FS	1:1522	1:2481
FSC	1:7666	1:16,816
FSX	1:68,995	-
FE	-	1:151,347
FC	1:34,497	1:21,621
F-	1:103,492	1:21,621
FAS	1:49	1:100
FAC	1:394	1:655
FAE	1:2275	1:2259
FAD *	1:2587	1:7567
FAO *	1:4600	1:75,674

* Variant not screened by the method used in Centre Hospitalier Universitaire de Liège before 2014. Neonates homozygous for Hb S or compound heterozygous for Hb S and β-thalassemia (FS) or compound heterozygous for Hb S and C (FSC) or another Hb variant (FSX). Neonates homozygous for Hb C or compound heterozygous for Hb C and β-thalassemia (FC). Neonates homozygous for Hb E or compound heterozygous for Hb CE and β-thalassemia (FE). Neonates with absence of Hb A (F-). Neonates likely heterozygous for a hemoglobin variant i.e., Hb S (FAS), Hb C (FAC), Hb E (FAE), Hb D-Punjab (FAD), Hb O-Arab (FAO).
